# The landscape of non-coding RNAs in the immunopathogenesis of Endometriosis

**DOI:** 10.3389/fimmu.2023.1223828

**Published:** 2023-08-22

**Authors:** Mohammad Abbaszadeh, Mohammadreza Karimi, Samira Rajaei

**Affiliations:** Department of Immunology, School of Medicine, Tehran University of Medical Sciences, Tehran, Iran

**Keywords:** endometriosis, inflammation, non-coding RNAs, miRNAs, lncRNAs, siRNAs, CircRNAs

## Abstract

Endometriosis is a complex disorder that is characterized by the abnormal growth of endometrial-like tissue outside the uterus. It is associated with chronic inflammation, severe pelvic pain, infertility, and significantly reduced quality of life. Although the exact mechanism of endometriosis remains unknown, inflammation and altered immunity are considered key factors in the immunopathogenesis of the disorder. Disturbances of immune responses result in reduced clearance of regurgitated endometrial cells, which elicits oxidative stress and progression of inflammation. Proinflammatory mediators could affect immune cells’ recruitment, fate, and function. Reciprocally, the activation of immune cells can promote inflammation. Aberrant expression of non-coding RNA (ncRNA) in patient and animal lesions could be suggestive of their role in endometriosis establishment. The engagement of these RNAs in regulating diverse biological processes, including inflammatory responses and activation of inflammasomes, altered immunity, cell proliferation, migration, invasion, and angiogenesis are widespread and far-reaching. Therefore, ncRNAs can be identified as a determining candidate regulating the inflammatory responses and immune system. This review aims in addition to predict the role of ncRNAs in the immunopathogenesis of endometriosis through regulating inflammation and altered immunity based on previous studies, it presents a comprehensive view of inflammation role in the pathogenesis of endometriosis.

## 1 Introduction

In 1860, Carl Freiherr von Rokitansky proposed endometriosis (EMS) as a chronic gynecological disorder defined by endometrial glands and stroma outside the uterus cavity (1). The disease has heterogeneous nature in terms of manifestations and types of lesions. For example, it can be grouped into three phenotypes: superficial peritoneal lesion (SUP), cysts in the ovaries or endometriomas (OMA), and nodules with depth >5mm, known as a deep infiltrating lesion (DIE). SUP is the least severe form of endometriosis; whereby superficial lesions of the endometrium develop on the peritoneum covering the pelvic cavity. OMA presents as cystic masses that arise from ectopic endometrial tissue within the ovaries. While SUP and OMA are common types of endometrioses, DIE is characterized by lesions that penetrate tissues. This form of endometriosis is implicated as a severe form of endometriosis (2). Although 20^_^25% of patients with unknown symptoms are remained undiagnosed, approximately 10-15% of women their reproductive age might develop the disease (3, 4).

The exact origin and pathophysiology of endometriosis have yet to be known entirely. There are several yet to fully confirmed theories that describe endometriosis pathogenesis (5). These theories have claimed that those lesions might be originated from the uterine endometrium or tissues other than the uterus (6). Sampson’s theory, the most widely accepted hypothesis, shows retrograde menstruation can occur when the endometrium detaches during menstruation and backflow into the pelvic cavity through the fallopian tubes. The tissue fragments in this retrograde blood flow are implanted on the peritoneal and ovarian surfaces and fail to be cleared (7). Although retrograde menstruation occurs in more than 90% of women, only 10% suffer from endometriosis (8). Thus, genetic and epigenetic, inflammatory milieu, impaired immunity, and female sex hormones are necessary to promote cell survival, proliferation, lesion formation and maintenance (5).

Notwithstanding the multifactorial causes of endometriosis and its multifaceted and complex nature, the immune system is essential in detecting and eliminating abnormally growing tissue, including ectopic endometrial lesions (9). In healthy women, lesions are cleared by immunosurveillance. However, in women with endometriosis, altered immunity (i.e., impaired macrophage and natural killer (NK) cell activity, T-helper1 (Th1)/T-helper2 (Th2) imbalance, and lymphocyte T (Th1) dysfunction) reduce the elimination of endometrial cells, and therefore endometrial cells implant and lesions develop (9, 10). In addition to increasing oxidative stress, implants are considered a foreign element triggering an immune response and the gradual development of inflammation (9). In the immunopathogenesis of endometriosis, inflammation resulted in pain, tissue remodeling, lesion formation, fibrosis, and infertility (11). Aberrant production and secretion of proinflammatory mediators such as cytokines, chemokines, prostaglandins, and metalloproteinases, as well as alteration of fate, function, and infiltration of immune cells in sites of lesions are some changes involved in this disrupted response (12–14). Therefore, inflammation and altered immunity are central to the pathogenesis of endometriosis.

Human genome studies have pinpointed unique genomic areas significantly associated with endometriosis risk (15). Most of these regions are found in non-coding introns and intergenic regions. Non-coding RNAs (ncRNAs) are functional RNAs mostly transcribed from these sites of DNA and constitute fully connected layers of internal signals that regulate several processes in gene expression (16). Non-coding RNAs which lack protein-encoding ability are categorized based on their size (length), structure, and function (17). Short and long ncRNAs are two subclasses of ncRNAs that are differentiated by their length. Generally, transcripts larger than 200 nucleotides are long non-coding RNAs (lncRNAs) compared to short non-coding RNAs with less than 200 nucleotide length, like the ~ 22-nucleotide-long microRNA(miRNA). ncRNAs regulate gene expression at the transcriptional and post-transcriptional levels by manipulating gene expression procedures, epigenetic modification, and RNA processing (17, 18). Considering the principal role of both inflammation and disturbed immunity in the progression of EMS, this study aims to summarize the potential role of these transcripts in EMS pathogenesis through the regulation of inflammation and altered immunity.

## 2 Research methodology

This study considers inflammation and disturbed immunity as the two main contributing factors to endometriosis progress. Thus, we first focus on these mechanisms in endometriosis progression.

### 2.1 Inflammation and altered immunity

**Figure 1:**
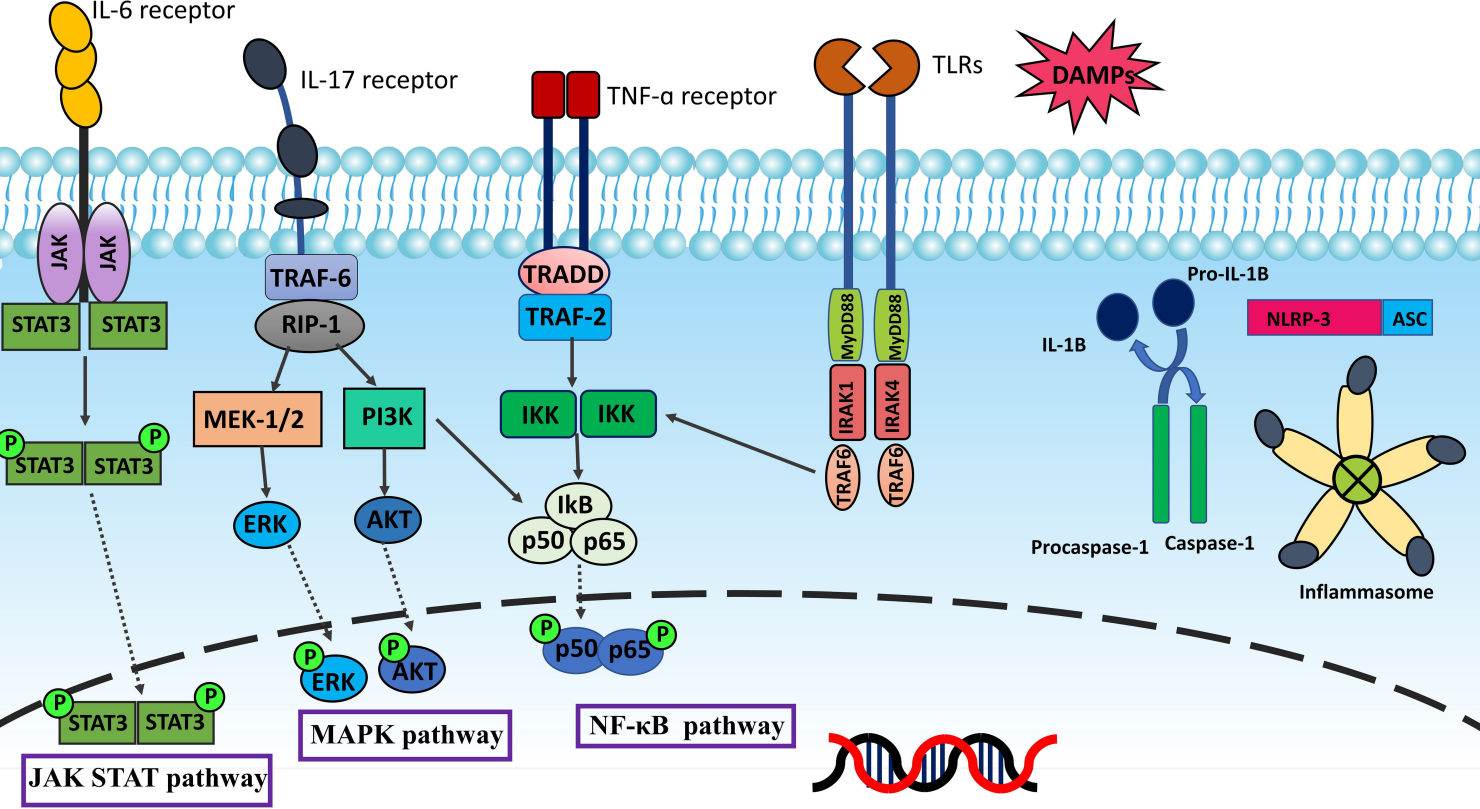
Inflammation is mainly regulated by NF-κB, MAPK, and JAK-STAT pathways. TLRs and inflammatory cytokines such as TNF-α and IL-17 activate NF-κB pathway via IκB kinase (IKK). The proteasome degradation of phosphorylated IκB releases NF-κB for nuclear translocation and gene transcription activation. The MAPK pathway provides intracellular signaling triggered by external stimuli by phosphorylating and activating MAPK kinases like MEK1/2. MAPK kinases phosphorylate various proteins, including transcription factors like ERK1/2, and phosphorylated ERK translocate to the nucleus resulting in the transcription of inflammatory mediators. Upon IL-6 binding, the IL-6 receptor transduces the signal to activate JAKs, then JAKs phosphorylate the receptor. The phosphates are subsequently bound by two STAT proteins, which JAKs then phosphorylate to create a dimer. The dimer penetrates the nucleus, binds to DNA, and causes target genes to be transcribed. When PAMPs and DAMPs trigger the innate immune system and inflammatory signaling, NLRP3 forms oligomers and activates caspase-1. This starts the processing and release of IL-1β, which is a pro-inflammatory cytokine. Inflammasomes are sensors of the innate immune system that regulate the activation of caspase-1 and generate inflammation. As DAMPs stimulate the innate immune system, NLRP3 starts oligomerization and activation of caspase-1, causing the release of the pro-inflammatory cytokine IL-1β.

Inflammation is one of the underlying factors which causes infertility and pelvic pain in patients with endometriosis (19). The possible mechanism which explains why endometriosis comes along with inflammation is that endometriotic lesions like normal endometrium can respond to cyclic variations of estrogen and progesterone in menstruation. However, unlike normal endometrium, patients are unable to shed degenerating endometriotic tissue during menstruation. Subsequently, blood builds up inside endometriotic lesions which causes the fenton reaction and Reactive oxygen species (ROS) production. This situation results in the degradation of some endometriotic cells and releasing of Damage-associated molecular patterns (DAMPs), triggering proinflammatory signaling pathways, which ultimately activate the production of inflammatory mediators (20, 21). There are three primary inflammatory pathways, Nuclear factor kappa B (NF-κB), Mitogen−activated protein kinase (MAPK), and Janus kinase/signal transduction and activator of transcription (JAK-STAT), that control the pathogenesis of endometriosis (22, 23). NF-κB is a paramount regulator of inflammation and immunological response. Five related transcription factors make up the NF-κB family: p50, p52, RELA (p65), RELB, and c-Rel. In normal conditions, the presence of cytoplasmic inhibitor of kappa B (IκB) proteins inhibits NF-κB. Inflammatory stimuli employ signal transduction to activate IκB kinase (IKK). IKK modulates the activation of the NF-κB pathway by phosphorylation of IκB. The proteasome degrades phosphorylated IκB leading to the release of NF-κB for nuclear translocation and activation of gene transcription (24). **Figure 1** summarizes inflammatory signaling pathways.

Principal inflammatory mediators produced by these pathways, such as Cyclooxygenase-2 (COX-2), Interleukin-1 (IL-1), Interleukin-8 (IL-8), Tumor necrosis factor alpha (TNF-α), and Prostaglandin E2 (PGE2), mediate inflammation through interacting with their receptors and immune cells (25–27). COX-2, as a product of inflammatory pathways, regulates the progression of EMS by producing inflammatory mediators and differentiation of CD16- NK cells with low levels of cytotoxicity in the peritoneal fluids of EMS patients. This type of NK cell is advantageous for evading endometriotic lesions from immunosurveillance (28). COX-2 that produces PGE2 can promote cell proliferation and inhibit cell death by multiple transactivating complexes of the signaling pathways triggered by PGE2 and its receptors, prostaglandin E receptor 2 (EP2) and prostaglandin E receptor 4 (EP4) (29). In addition, PGE2 inhibits the ability of peritoneal macrophages (pMQs) to carry out phagocytosis because it reduces the expression of matrix metalloproteinase (MMP9) and the scavenger receptor (CD36). Furthermore, PGE2 can alter macrophage polarization and shift it towards M2, in which the cells have reduced phagocytosis potential against displaced endometrium and enhanced ability to induce fibrosis and angiogenesis in endometriotic lesions (30–32).

**Figure 2:**
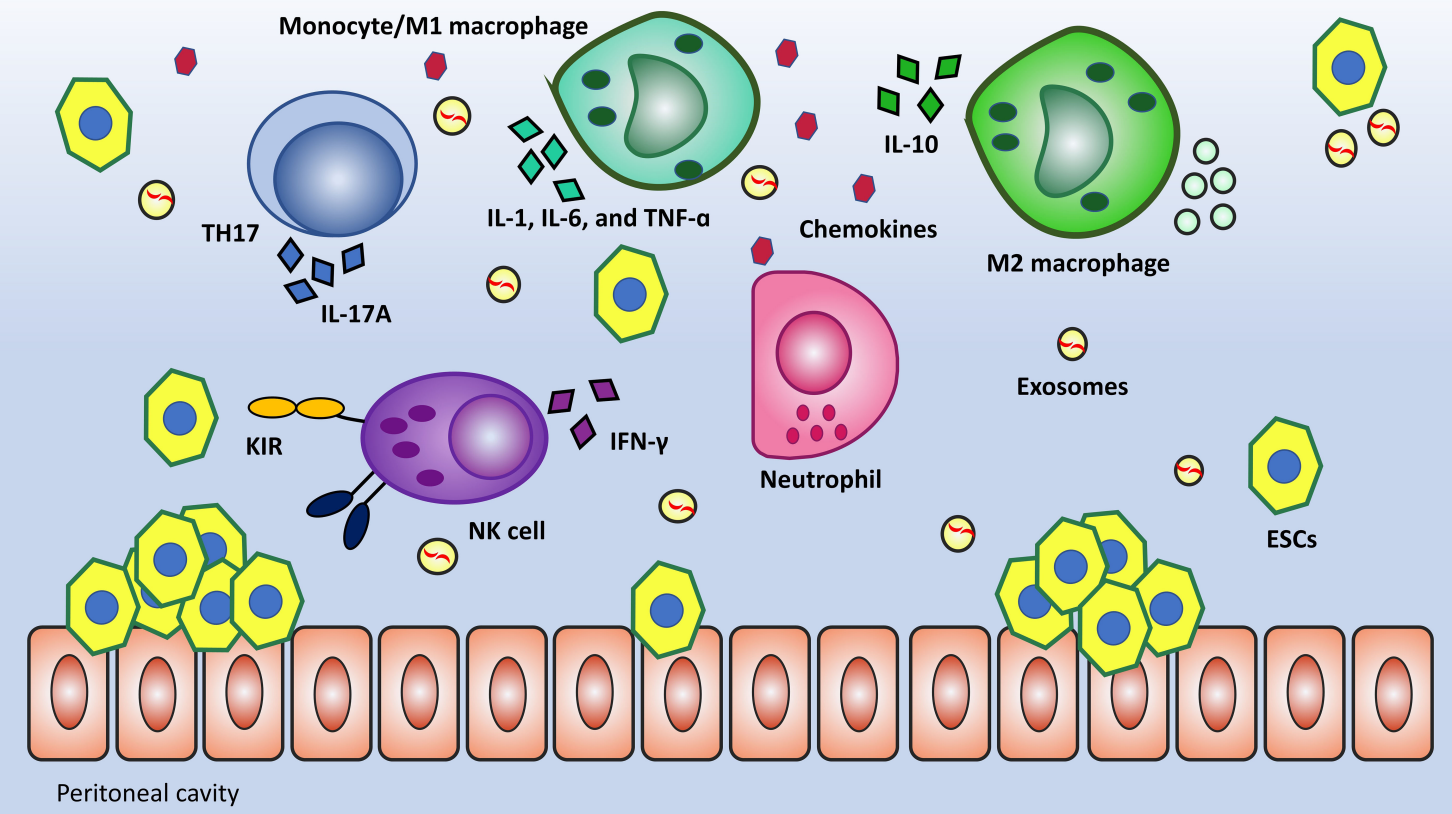
Local and systemic alteration of immunity is an outstanding feature of EMS pathogenicity. Macrophages have dual functions in tissue injury and repair. M1 by the secretion of proinflammatory mediators (TNF-α, IL-6, and IL-1) and M2 by the secretion of anti-inflammatory cytokines (IL-4, IL10, and IL-13) have been suggested to inhibit and promote the development of endometriosis, respectively. TH17 is a subset of differentiated T cells that has remarkably increased in number. These cells can secrete IL-17A having a synergistic effect with TNF-α in terms of inflammation. One further change in immunity is low-level NK cells’ cytotoxicity against ESCs due to the upregulation of inhibitory receptors like KIR2DL1.

Multitude studies showed that patients with severe endometriosis had impaired local and systemic immunity, including impaired macrophage phagocytosis, T cell activation, and NK cell activity (33, 34). The majority of immune cells in the peritoneal cavity are Macrophages, their defensive function is mainly demonstrated via phagocytosis and cytokine secretion. The process of phagocytosis primarily developed to clear cellular debris. This mechanism experiences drawbacks in EMS due to the downregulation of CD36 and MMP9, which are substantially involved in the clearance of cellular debris (35). In the case of lymphocytes, the Th1/Th2 Imbalance in EMS has been described as skewed toward a Th2 immune response, but recently, more attention has been paid to the role of T-helper 17 (TH17) and Regulatory T-cells (Tregs). TH17 releases Interleukin-17A (IL-17A), which synergistically boosts the secretion of IL-8 and COX-2 when accompanied by TNF-α. Moreover, if Th17 cells dominate Tregs, debris clearance will be more effective because of Interleukin-6 (IL-6) and IL-17 inducing inflammation (36–38). Another cell that directs attention toward itself is the NK cell can kill the target cell without prior sensitization. Several studies have shown that cytotoxic mediators (perforin and granzyme) and cytokine Interferon gamma (IFN-γ) release in response to effector molecules is diminished in EMS women (39, 40). Evaluation of peritoneal NK cell surface markers revealed that killer cell immunoglobulin like receptor, two Ig domains and long cytoplasmic tail 1 (KIR2DL1 or CD158a), an inhibitory KIR, was considerably upregulated in women with endometriosis compared to women without the condition. KIR2DL1 (CD158a) inhibits NK cell cytotoxicity by transferring an inhibitory signal (41, 42). **Figure 2** provides a summary of altered immunity in EMS.

Peritoneal cytokines and chemokines, synthesized by immune, mesothelial, and ectopic endometrial cells, interact locally and systemically in EMS patients. Various cytokines, including IL-1, IL-6, IL-8, interleukin (IL-10), and TNF-α, were elevated in the peritoneal fluid of EMS-affected women (43) (44, 45). There is some evidence that these cytokines contribute to macrophage activation, inflammatory responses, and increased angiogenesis (46). Chemokines or chemoattractant cytokines which mediate leukocyte migration, have been categorized into two major groups in EMS (47). CCL5, CCL2, and CCL11 are CC-chemokine ligands that target monocytes (48), T lymphocytes (49), and eosinophils (50). CXC-chemokine ligands (such as CXCL1, CXCL2, CXCL8, CXCL5, and CXCL12) attract monocytes and neutrophils to improve inflammation by enhancing immune cell trafficking (51).

The expression profiles of ncRNAs vary depending on the kind of cell, the stage of development, and the physiological conditions to which the cells are exposed (52). This holds true even for immune system cells; fluctuations in the expression levels of ncRNAs in immune cells have been observed in various conditions, such as cancer, inflammation, and autoimmune diseases (53, 54). ncRNAs are regulators of immune system function because of their ability to alter gene expression in immune cells. Precisely, ncRNAs control immune cell differentiation, activation, proliferation, cytokine generation, and migration. Moreover, ncRNAs can modulate the expression of genes involved in inflammation by acting as either positive or negative regulators (55). As ncRNAs have become indispensable transcriptional regulators during the immune response (56, 57). This review will focus on the biology of ncRNA in the immunopathogenesis of EMS.

## 3 Non-coding RNAs have roles in the inflammation and immunopathogenesis of EMS

### 3.1 miRNA

miRNAs are potent regulators of a variety of physiological or cellular processes that occur in EMS, including cell differentiation, proliferation, migration, and inflammation (58). One of the mechanisms called sponging microRNAs modulate gene expression due to their sequences interacting with the 3′-end, and less frequently the 5′-end, of mRNA generated from target genes (59). By comparing eutopic and ectopic endometrium, endometriomas, peritoneal fluid, and blood from patients with EMS, numerous studies have identified that miRNAs have a differential expression during EMS (60, 61). This ncRNA has the potential to be a diagnostic biomarker for the EMS. However, no specific miRNA has been transmitted from the laboratory to the clinic for diagnostic purposes in EMS (18, 62). Although being a diagnostic biomarker has been studied more, miRNAs can control the progression of the disease by affecting various determining mechanisms.

#### 3.1.1 miRNA and NF-κB

Some research has suggested that miRNAs in endometrial tissue might affect the expression of inflammatory mediators (63–66). miR-199a and miR-16 for example known to repress COX-2 translation, were downregulated in EMS (63, 64). Dai et al. reported that the downregulation of IKK protein by miR-199a was associated with both decreased IκB-α phosphorylation and lowered NF-κB nuclear translocation. In consequence, miR-199a may inhibit NF-κB pathway activation by the downregulating inhibitor of nuclear factor kappa-B kinase (IKKβ) protein expression. IL-8 mRNA and protein, which promotes adhesion, invasion, and proliferation of endometrial stromal cells (ESCs), were also downregulated following transfection with miR-199a (63, 67). Therefore, the decrease of miR-199a expression in EMS might be related to the abnormal activation of NF-κB in ectopic endometrial stromal cells (eESCs). Research conducted by Wang et al. in 2020 demonstrated that the microRNA known as miR-16 could inhibit ESCs migration and invasion by blocking the NF-κB signaling pathway (64). Although the role of this microRNA in inflammation was not directly studied in this research, it appears that inhibiting NF-κB kinase subunit (IKK) can block the NF-κB pathway, resulting in a decrease in the production of inflammatory mediators. Dai and Wang et al. demonstrated activation of the NF-κB pathway can stimulate the proliferation and invasion of endometrial cells and the production of inflammatory cytokines. Downregulation of miR199a and miR-16 can cause activation of NF-κB due to they are unable to block IKK. Downregulation of These miRNAs can be used as a biomarker for EMS patients as well as detection of Hepatocellular Carcinoma patients (68).

In the endometriosis mice model, according to a study by ZHANG et al., the expression level analysis of miR-138 and CD11b showed a significant decrease in EMS compared to the control group. When the uterine endothelial cells were co-cultured with THP-1 cells, miR-138 expression was subsequently downregulated, which caused an increase in inflammation. In contrast, mimic transfection of miR-138 declined inflammation in uterine endothelial cells by upregulating miR-138. In addition, when uterine endothelium and THP-1 cells were cocultured, downregulation of miR-138 enhanced the expression of the NF-kB in THP-1 cells, which can strengthen inflammation as an important source of secreting cytokines (65). miR-138 was examined in THP-1 and uterine endothelial cells while they were cocultured. In both cell types, the decrease in miR-138 resulted in an escalation of inflammation. In this study, the NF-κB pathway was investigated, which was found to be activated in THP-1 cells. While this is not the only pathway through which miR-138 can exert its effects (69), it is advisable to investigate the AKT pathway in future studies as well.

Another miRNA known as miR-182 can inhibit the NF-kB pathway, inflammation, migration, and invasion by targeting RELA (p65). To study the functions of miR-182 and RELA in endometriosis, they first assessed their expression levels in normal and ectopic endometrium. The results of the qRT-PCR study demonstrated that the level of miR-182 continued to decline when the condition changed from normal to eutopic and ectopic. Nevertheless, RELA expression level, similar to COX-2, increased concurrently with this process. This inverse relationship is repeated in western blot analyses at the protein level. Eventually, miR-182 mimic and miR-182 inhibitory plasmid transfection in eESCs resulted in a fall and a rise in inflammatory mediators, respectively (66). Therefore, by targeting RELA and altering the expression level of its downstream protein (COX-2), the decrease and increase of miR-182 in eESCs result in an increase and a decrease in inflammation in the endometriosis microenvironment, respectively.

#### 3.1.2 miRNA and alternative inflammatory pathways

Some microRNAs have the potential to exert an indirect influence on the NF-κB pathway as well as other pathways (70–72). According to Lin et al., endometriotic lesions contain stromal cells with elevated miR-20a levels. The overexpression of miR-20a causes a decrease in the expression of phosphatase, which is known as Dual-specificity phosphatase-2 (DUSP-2), which leads to the prolonged activation of Extracellular-signal-regulated kinases (ERK) and Hypoxia-inducible factor-1α (HIF-1α) axis. This activation can improve PGE2 production, contributing to inflammation (70). In addition to its potential to induce inflammation through the HIF-α pathway, miR-20a can also enhance the production of PGE2. Based on the previously described functions of PGE2 on immune cells, such as macrophages (30) and NK cells (28), further studies can be conducted in order to determine how PGE2 affects immune cells downstream of HIF-α.

Within the endometriosis mouse model, let-7b functioned as a therapeutic miRNA. After treatment of mice with let-7b, gene expression analysis in ESCs showed a decline in genes accounting for the expansion of ESCs and Toll-like receptor 4 (TLR4) and IL6 in the let-7b treatment group compared to controls, suggesting that inflammation associated with EMS may be mitigated by this treatment (72). Parallel to this study, another investigation showed that the downregulation of let-7b and overexpression of miR-125b-5p increase the production of proinflammatory mediators in the serum of EMS patients compared to controls. This elevation of inflammatory mediator happened while macrophages transfected with a miRNA-125b mimic or a let-7b inhibitor could promote and suppress the expression of miR-125b-5p and let-7b, respectively (71).

Recent investigations into miR-215-5p found the differential expression of serum exosomal of this miRNA, miR-26b-5p, and miR-6795-3p compared to the control by microarray analysis. According to the KEGG database, identified target genes primarily engage in the MAPK and PI3k-AKT signaling pathways (73). Also, another predicted target gene found in this study for miR-215-5p was CXCL2. CXCL2 is a chemokine with a potent chemotactic impact on neutrophils and monocytes. Thus, it appears that low expression of miR-215-5p leads to high levels of CXCL2 expression in EMS patients, which may increase inflammation and monocyte and neutrophil recruitment to the abdominal cavity (74, 75).

Serum miR-17 is a diagnostic marker for endometriosis. The correlation analysis showed it is inversely linked with IL-4 and IL-6 receptors. Bioinformatics anticipated that miR-17 would target the 3’-UTR of IL-4 and IL6 receptors in separate places. These findings indicate that decreasing miR-17 expression may boost IL-4 and IL-6 receptors, allowing the specific cytokines to exert their effect more efficiently in this condition (76). MicroRNAs can have an impact on inflammation independent of the NF-κB pathway. For instance, the exacerbation of inflammation is caused by the low level of some microRNAs, such let-7b and miR-215-5p and the high level of some others, such as miR-20a and miR-125-5p, in endometriotic individuals compared to the control group. As demonstrated in studies, the HIF-α pathway, MAPK, PI3K, Toll-like receptor 4, cytokines, and interleukin 4 and 6 receptors are involved. However, in some of these studies, such as the research conducted by Wu Y et al., the effects of suppressing and increasing pathological microRNAs in the invivo phase have not been examined, which can propose new therapeutic approaches for these patients.

**Figure 3:**
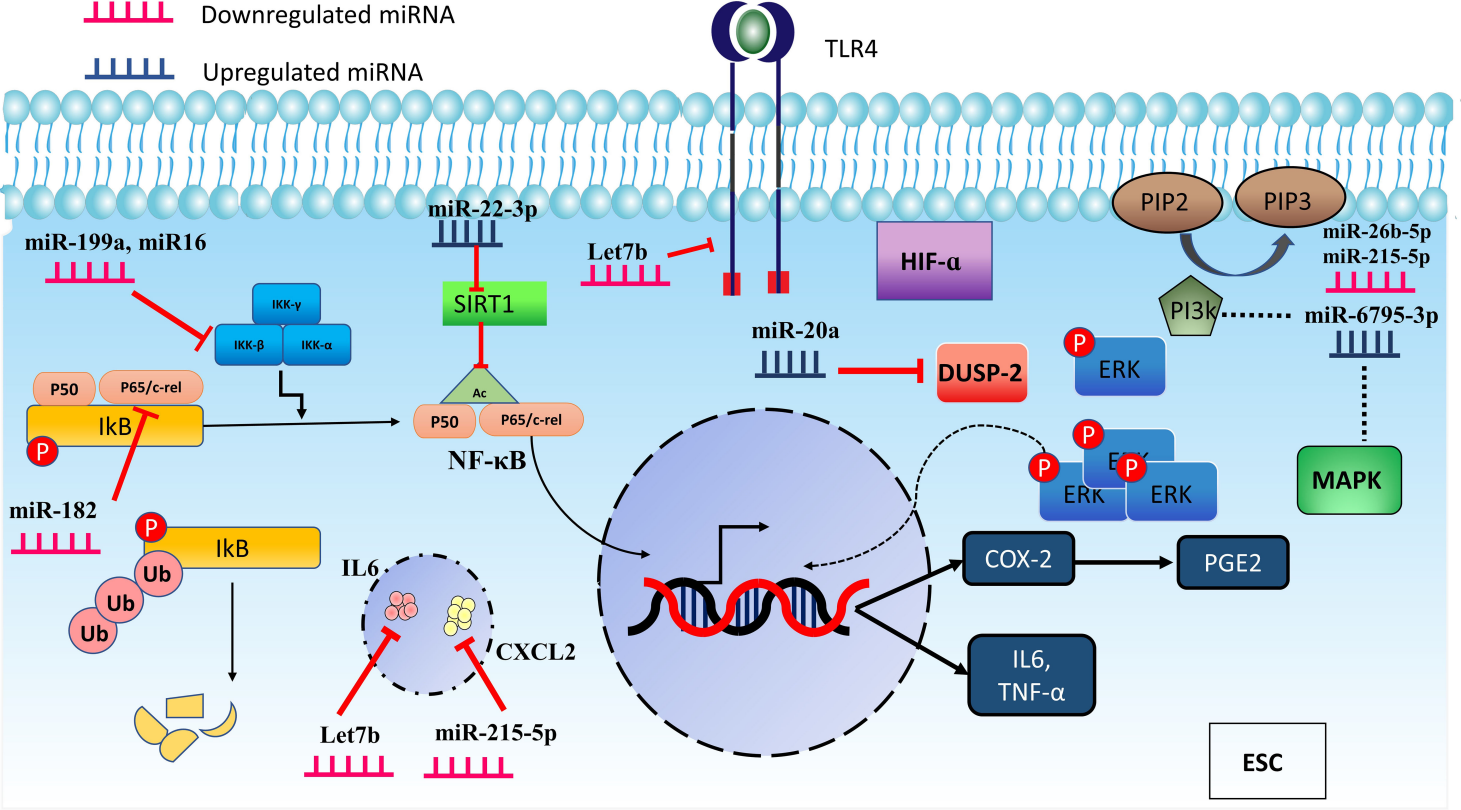
MicroRNAs are involved in regulating inflammation through different pathways. miR-16, miR-199a, and miR-182 downregulation induce inflammation in ESC by modulating the IKKβ/NF-κB pathway. In addition, miR-22-3p can increase inflammation by impacting the SIRT-1 which acetylates p65, causing p65 to move more efficiently to the nucleus and boost the transcription of inflammatory mediators. Furthermore, some miRNAs act through other pathways. miR-20a leads to the prolonged activation of ERK and promotes inflammation by suppressing DUSP-2. MAPK and PI3K are also affected pathways by miR-26b-5p, miR-6795-3p, and miR-215-5p which can participate in inflammation as prominent pathways.

Proinflammatory mediators, including IL-1B, TGF-β1, and TNF-α, indirectly reduce chicken ovalbumin upstream promoter transcription factor II (COUP-TFII) in ESCs via miR-302a. COUP-TFII inhibits transcription of the COX-2 gene by binding directly to its promoter. This mechanism appears to be positive feedback for increasing inflammatory response by the mediation of miR-302a (77). **Figure 3** displays miRNAs having a role in inflammatory pathways.

#### 3.1.3 miRNA and altered immunity

##### 3.1.3.1 miRNA and macrophage

**Figure 4:**
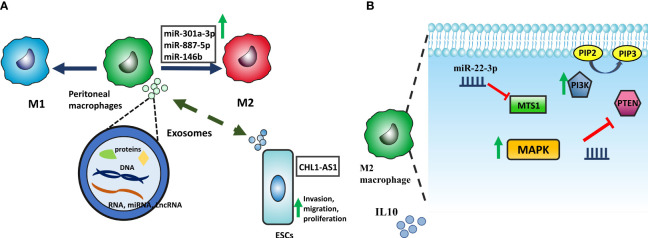
**(A)** ESCs and macrophages have mutual interaction by exosomes containing protein, RNA, and DNA. CHL1-AS1 lncRNA transposed from pMQs promotes invasion, migration, and proliferation by accumulating MDM2. Moreover, exosomes derived from ESCs can transfer miRNAs, including miR-146b, miR-301a-3p, and miR-887-5p which alter macrophage polarization into M2. **(B)** Upregulation of miR-301a-3p affects MTS1 expression and reduces it. With the reduction of MTS1, MAPK signaling is activated, resulting in the secretion of the anti-inflammatory factor IL-10. miR-887-5p is also involved in macrophage polarization by promoting of the PI3K signaling pathway. PTEN is a phosphatase inhibiting PI3K to produce Phosphatidylinositol (3,4,5)-trisphosphate (PIP_3_); by reduction of this phosphatase via miR-887-5p, PI3K signaling pathway acts more and skew macrophage into M2 phenotype.

Dysregulation of macrophage activity can significantly contribute to the development and progression of EMS since macrophages are intimately involved in initiating an inflammatory response, wound healing, and tissue homeostasis (78). Animal studies have revealed correlations between macrophage polarization imbalances and the development of lesions (79). Changes in endometriosis-related macrophage phenotype may be due to the peritoneal cavity’s microenvironment and reflected endometrial cells. There is reciprocal communication between macrophages and these factors, mediated by cytokines and exosomes (80). Exosomes can enter cells and release proteins, mRNA, lncRNA, and miRNA into the cytoplasm and then participate in various cellular processes, such as the polarization of macrophages and cytokine production (81, 82). Exosomes derived from eESCs upregulate the expression of CD163, a specific marker of M2 macrophages, in pMQs from EMS patients. These exosomes can potentially transfer the microRNA miR-301a-3p into pMQs, which could lead to an upregulation of phosphoinositide 3-kinases (PI3K) expression and downregulation of phosphatase and tensin homolog (PTEN), a phosphatase inhibiting PI3K to produce Phosphatidylinositol (3–5)-trisphosphate (PIP_3_) from Phosphatidylinositol 4,5-bisphosphate (PIP2), this alteration in PI3K signaling pathway ultimately shifts polarization of the macrophages into M2 (83). Furthermore, miR-887-5p is another ncRNA that promotes the M2-type polarization of macrophages and the secretion of IL10 by activating the MAPK pathway. It is well-established that peritoneal fluid or medium from cultured peritoneal macrophages of endometriosis patients had considerably higher IL-10 protein levels. IL-10, an anti-inflammatory cytokine, is vital for limiting autoimmunity and inflammatory responses (84, 85) (**Figure 4**). Taken together, exosomes containing miR-301a-3p and miR-887 can alter pMQs polarization towards M2 via PI3K and MAPK signaling pathways, respectively.

Patients with EMS who reported pain also showed higher miR-146b than those who reported no pain. miR-146b is implicated in the negative regulation of inflammation through downregulating polarization of pMQs toward M1. In addition, macrophages derived from individuals who carried the CT/CC genotype of the miR-146b rs1536309 gene showed decreased expression of the miR-146b gene and an increased capacity for producing inflammatory cytokines (86).

pMQs can modulate the capacity of eESCs in the proliferation, migration, and invasion via upregulated NF-κB expression. Exosomal miR-22-3p derived from pMQs is transported to eESCs (87). MiR-22-3p by targeting and reducing SIRT1, a NAD+dependent class III histone deacetylase that regulates the NF-κB pathway (88), promotes the proliferation, migration, and invasion of eESCs via upregulated NF-κB expression. Additionally, it can be hypothesized that activating the NF-κB pathway in eESCs also improves the production of inflammatory mediators.

Recent research on human endometriotic cell lines demonstrates that inhibition of EP2 and EP4 pharmacologically can alter the expression pattern of microRNAs. According to Ingenuity Pathways Analysis (IPA) was performed on differentially expressed miRNAs in endometriotic epithelial (12Z) and endometriotic stromal (22B) cells treated with an inhibitor of EP2 and EP4, miR-34c-5p and miR142-3p have a role in the inflammatory response, as do act in macrophage phagocytosis malfunctions (89).

miR-451a has emerged as powerful factor in elevating phagocytosis by rearrangement of the cytoskeleton (90). Although this miRNA was shown which is reduced in eutopic endometrium of EMS patients (91), it has not been investigated in macrophages in EMS individuals. In general, in endometriosis studies, more attention has been given to the role of ncRNAs in macrophage polarization. Additionally, the miR-142-3p investigated in endometriosis studies and its role in disrupting antigen presentation by primary macrophages can attract attention to be investigated in future research (92, 93). Although phagocytosis (94) and antigen presentation (78) are actions performed by macrophages during this disease, additional investigations using imaging techniques, suppression and upregulation of ncRNAs can shed light on the examination of cellular remodeling and migration of these cells.

##### 3.1.3.2 miRNA and NK cells

Several investigations have revealed that women with endometriosis have NK cell malfunction, which leads to the immunological escape of fragments refluxed into the peritoneal cavity (95, 96). In terms of how miRNAs can influence NK cell cytotoxicity, miR-20a not only functions in inflammation but also can improve NK cytotoxicity by inducing perforin. It is hypothesized that due to the role of this microRNA in increasing the cytotoxicity of NK cells, unlike ESCs, the amount of this microRNA has decreased in NK cells. This drop in expression may be a factor in the functional defect of these cells (97). Moreover, the putative role of miR-182 in NK cytotoxicity can also be highlighted. It has been demonstrated in the research conducted by Abdelrahman on patients with hepatocellular carcinoma that the enhancement of NK cell cytotoxicity can be caused by miR-182, which indirectly mediates a complex modulation of the Natural Killer Group 2D (NKG2D) and The Natural Killer Group 2A (NKG2A) receptors levels in NK cells (39, 66, 98, 99). Future studies require to analyze whether aberrant expression of this miRNA in NK cells can be a potential for failure of NK cells cytotoxicity in EMS.

Unlike other studies, NK cell deficiency was examined in connection with ESCs in the Jie Mei et al. study. In this research, they found that the amount of autophagy in eESCs is lower than in normal cells, and this change in the amount of autophagy causes an increase in the expression of IL-8 and IL-23A. When NK cells were cocultured with lowered autophagy endometrial cells, it was demonstrated that the decrease in the amount of MIR1185-1-3p in NK cells causes an increase in COX-2 and PGE2 which can differentiate low granzyme, perforin, and IFN-γ NK cells (28). **Table 1** shows up- and down-regulated miRNAs in the endometriotic samples playing a role in immune system response.

**Table 1:** differentional expressed miRNA having roles in immune system response in endometriosis.

miRNA	Species	Sample	Assessed cell line	Target regulator	Signal pathway	Mechanism	Study
**miR-199a↓**	Human	Ectopic and eutopic endometrium from ovarian endometriomas patient(n=12) and control(n=12)	_	IKKβ	NF-κB	miR-199a is downregulated in EMS patient; therefore, with the upregulation of IKKβ, the NF-κB pathway is more active.	(63)
**miR-16↓**	Human	Ectopic and eutopic endometrium from patient(n=20) control(n=20)	_	IKKβ	NF-κB	Downregulation of miR-16 can contribute to the activation of the NF-κB pathway.	(64)
**miR-138↓**	Mouse	Peritoneal fluid from mice model of EMS	THP-1, ESCs cell line	P65	NF-κB	Downregulation of miR-138 in THP-1 cocultured with uterine endothelial cells increases the NF-κB pathway in THP-1.	(65)
**miR-182↓**	Human	Ectopic and eutopic endometrium from EMS patients(n=20) and Normal endometrium(n=20)	_	RELA	NF-κB	miR-182 inhibits the NF-κB signaling pathway by directly targeting RELA	(66)
**miR-20a↑**	Human	Endometriotic lesions from pelvic endometriosis and ovarian endometrioma in patient with EMS (n=31) and normal endometrium from free EMS patients (n= 17), and 12 sets of paired eutopic and ectopic endometrial tissues from patients with EMS	_	DUSP-2	HIF-1α	miR-20a, inhibiting DUSP-2, can provide prolonged activation of phosphorylated ERK, which raises PGE2 expression level and inflammation.	(70)
**Let-7b↓**	Mouse	Endometriotic lesions were removed from peritoneum, patient sample(n=5), control sample(n=5)	_	ER‐α, ER‐β, Cyp19a, KRAS 4A, KRAS 4B and IL‐6	IL6, TLR4	Treatment of mice with let-7b decreases inflammation by downregulation of IL6 and TLR4.	(71, 72)
**miR-26b-5p↓, miR-215-5p↓, and miR-6795-3p↑**	Human	Serum samples were collected from patients with ovarian EMS lesions in stage 1 and 2 based on rASRM(American Society of Reproductive Medicine (n=12), stage 3-4(n=30), control sample(n=24)	_	PTEN, CXCL2	MAPK and PI3k-AKT	Regulating inflammation via MAPK and PI3k-AKT signaling pathways.	(73)
**miR-17↓**	Human	Serum samples from EMS patient(n=80) and control(n=60)	_	_	_	Downregulation of miR-17 By affecting IL4 and IL6 receptors could induce inflammation	(76)
**miR-302a↑**	Human	Human endometrium samples from EMS patient(n=21) and control(n=36)	_	COUP-TFII	_	The aberrant expression of miR-302a by reduction of COUP-TFII and elevation of COX-2 can increase inflammation	(77)
**miR-301a-3p↑**	Human	ectopic endometrial tissues and normal human serum	THP-1	PTEN	PTEN-PI3K	Induction of M2 macrophage via PTEN-PI3K axis.	(83)
**miR-887-5p↑**	Human	Peritoneal fluid, eutopic and ectopic endometrium	THP-1	MTS1	MAPK	Induction of M2 macrophage via MTS1-MAPK axis.	(84)
**miR-146b↑**	Human	Endometrial tissue from patient with EMS(n=73), normal control(n=23), macrophages were extracted from peritoneal fluid	THP-1	IRF5	_	Overexpression of miR-146b can lead to the reduction of IRF5 and subsequently decrease polarization of M1 macrophage.	(86)
**miR-22-3p↑**	Human	pMQs were extracted from PF, and ESCs were isolated from ectopic endometrium in EMS patient(n=20)	_	SIRT1	NF-κB	miR-22-3p via targeting SIRT1 Increase NF-κB pathway in eESCs.	(87)
**miR-15a-5p↓, miR-367-3p↓, miR34c-5p↓, miR-141-3p↓, miR-142-3p↓, miR-598-3p↑, miR-193a-p3↑, miR-501-5p↑, and miR-518d-3p↑**	Mouse	Endometriotic lesions were taken from xenograft mice model of peritoneal endometriosis.	Immortalized endometriotic epithelial cell line 12Z and stromal cell line 22B	_	_	Based on IPA, noted microRNAs were engaged in the inflammatory response.	(89)
**miR-34c-5p and miR142-3p↑**	Mouse	Endometriotic lesions were taken from xenograft mice model of peritoneal endometriosis.	Immortalized endometriotic epithelial cell line 12Z and stromal cell line 22B	_	_	Based on IPA, noted microRNAs were engaged in macrophage phagocytosis malfunctions.	(89)
**MIR1185-1-3p**	Human and mouse	In human samples, 75 endometriotic tissues and 165 control samples were obtained by laparoscopy. In addition, NK cells were purified from PFMC of both human and mouse.	_	PTGS2	_	Downregulation of the mentioned miRNA can induce differentiation of the low cytotoxic NK cells by increasing of COX-2 and PGE-2	(28)

“↑” symbol means the expression of the written non-coding RNA is upregulated.

“↓” symbol means the expression of the written non-coding RNA is downregulated.

“-” there is no information about that subject.

## 4 Short interfering RNAs

Similar to miRNA, small interfering RNA (siRNA), also known as short interfering RNA or silencing RNA, inhibits the expression of certain genes with complimentary nucleotides on their sequence. This noncoding RNA inhibits translation by decaying mRNA after transcription. The primary distinction between siRNAs and miRNAs is that the siRNAs are more specific and target a single mRNA, while miRNAs have numerous targets (100). However, no proof refutes the hypothesis that cells are can independently produce siRNAs for their reasons. Studies on siRNAs conducted in EMS have originated from exogenous siRNAs from a bench scientist or viral infection. Then their effects on the pathogenesis of the disease are analyzed by researchers. The siRNA knock-down of NF-κB in the chick embryo model of endometriosis reduces vascularization of the chick chorioallantoic membrane and enhances apoptosis in ectopic tissues (101). These findings suggest that the inflammatory NF-κB pathway promotes the survival of eESCs and angiogenic pathways essential for developing EMS lesions. Additionally, The use of siRNA to knock down CD36 in macrophages results in the loss of phagocytic function, which was also found in the pMQs of patients undergoing EMS (102). To explore how ESCs and pMQs interact during the development of EMS, Fumiko Itoh showed that by blocking STAT3 by siRNA, the proliferation of ESCs was inhibited when the cells were cocultured with M2 macrophages (103).

### 4.2 LncRNA

LncRNAs are a class of endogenous, non-protein coding RNAs which have recently attracted attention in the context of EMS (104). LncRNAs can act in gene regulation in various ways, such as guiding chromatin-modifying enzymes, constructing scaffolds for Ribonucleoprotein complexes, being a precursor into miRNAs, and sponging miRNA the most important way of interacting with other non-coding RNAs (105). LncRNA, through sponging miRNA, can form a multi-layer gene regulation model called Competitive endogenous RNA (ceRNA) which shows the interaction of lncRNA, circRNA, miRNA, and mRNA with each other (106). Whole-genome lncRNA expressions were initially analyzed using a Human lncRNA Expression Microarray in four patients with ovarian endometriosis. Compared to eutopic endometrial tissues, 948 lncRNAs, and 4,088 mRNAs were shown to be dysregulated in ectopic endometrial tissues (107). Furthermore, high-throughput sequencing technology showed in Liu et al. research that eutopic endometrium (1200 lncRNAs) and ectopic endometrial (695 lncRNAs) expression levels are altered in women with EMS compared to healthy women (108). Aberrant expression of lncRNAs plays many roles in the pathophysiology of EMS, particularly in immunologic circumstances which will discuss in detail.

#### 4.2.1 LncRNA and inflammation

MALAT-1 is a lncRNA that mediates inflammation by sponging miR-142-3p, the upstream regulator of CXCR7.The research by Kuailing Tan found that the overexpression of MALAT-1 led to the low miR-142-3p expression, which, in turn, increased CXCR7 and other proinflammatory mediators. MALAT-1 may have a role in EMS by controlling inflammation through the release of cytokines such as TNF-α, IL-1, and IL-6 (93). In several studies, such as Tan and Arosh et al., the reduction of miR-142-3p due to factors such as MALAT-1 and pharmacological inhibition of EP-2 and 4 has been mentioned (89, 93). Considering the important role of this microRNA in inflammation and macrophage phagocytosis (109), investigating function of this microRNA, and related lncRNA axes will be helpful. For example, in a study conducted on patients with inflammatory bowel disease (IBD), the MALAT-1 and miR-142-3p axis were found to be effective in increasing inflammation by reducing the level of Transforming growth factor beta (TGF-β) (110).

Another lncRNA that can impact inflammation via modulating innate immune defense pathways and Interferons (IFN) signaling is LINC00339 which has a tissue-specific nuclear expression pattern in endometriotic lesions. The Bioinformatic analysis estimates that this lncRNA also has a leading function in impaired immune regulation like inflammatory pathways in EMS patients. Overexpression of LINC00339 in endometrial cell lines induced expression level of Patterns Recognition Receptors (PRRs) and activation of Interferon Regulatory Factor (IRF), a mediator of cytosolic PRRs (111). Overexpression of PRRs can provoke inflammation; nevertheless, the upregulation of signal transducer and activator of transcription-1 (STAT1), STAT3, and interferon epsilon (IFNE) mRNA indicates that LINC00339 also influences other essential genes involved in inflammation. In addition to this research, prior studies had reported that LINC00339 might boost the expression of NLR family pyrin domain containing 3 (NLRP3) by using miR-22-3p (112), which has elevated expression in ESCs via transported exosomes. NLRP3 is capable of activating interleukin-1B and the inflammasome (113). In light of these studies, more research is required to comprehend the connection between LINC00339-miR-22-3p-NLRP3 and inflammasome in EMS patients. Overexpression of HOTAIR in the ectopic endometrium contributes to the proliferation, migration, and invasion of ESCs, and the angiogenesis of human umbilical vein endothelial cells (HUVECs). By using the miR-761/Histone deacetylase 1(HDAC1) axis and triggering STAT3-mediated inflammation via an exosomal contact, this axis can also include inflammation as one of its targets (114).

Li Jiang et al. revealed in a 2020 study that hsa-miR-182-5p, which controlled the downstream gene CHL1, Neural Cell Adhesion Molecule L1-Like Protein, was downregulated by lncRNAs, including LINC01018 and LINC01272. In eutopic endometrium, LINC01272 or SMIM25 played a function in the inflammatory response through IFN-γ and TNF-α in the NF-κB signaling pathway, as well as LINC01018 which has a role in the epithelial-mesenchymal transition pathway (115). Moreover, in an earlier investigation on hepatocellular cancer, forkhead box O1 (FOXO1) was determined as an additional target gene for the LINC01018/hsa-miR-182-5p axis. Forkhead box proteins (FOXOs) are a transcription factor family that regulates genes involved in cell proliferation, differentiation, apoptosis, and inflammation. In this study, LINC01018 minimized apoptosis in Hepatocellular carcinoma cells by inhibiting hsa-miR-182-5p and influencing the FOXO1 gene which has a positive correlation with proinflammatory signaling molecules (IL-1 and TNF-α) (116–118). Although researchers in EMS have not reported the correlation between LINC01018 and FOXO1, further exploration can be conducted in LINC01018/hsa-miR-182-5p/FOXO1 axis, which might induce endometrial inflammation.

#### 42.2 LncRNA and altered immunity

##### 4.2.2.1 LncRNA and macrophage

SMIM25 or LINC01272 which captures hsa-miR-182-5p often known as PELATON due to its role in macrophage phagocytosis (119). John Hung et al. discovered in the study on atherosclerotic plaques that depletion of LINC01272 caused a reduction in the expression of CD36 and, consequently, phagocytosis in macrophages, similar to what has been reported in EMS (102, 119). Thus, the LINC01272 expression level in pMQS might decline despite ectopic endometrium. This notion has to be verified by an additional study on LINC01272 in pMQS. The influence of lncRNAs on macrophage phagocytosis is only one aspect of their regulatory function and interaction. The expression level of the lncRNA CHL1-AS1 in EMS is increased by exosomes that are driven from pMQS and transferred to eESCs. This lncRNA exerts its effect via sponging miR-610, which interacts with the 3’-UTR of Mouse double minute 2 (MDM2). Sponging miR-610 resulted in MDM2 accumulation, which boosted eESC proliferation, migration, and invasion (120). Therefore, macrophages not only aid implantation of eESCs by reduced phagocytosis but also, through exosomes transferred from these macrophages to endometrial cells, they can enhance the proliferation and invasion of these cells.

##### 4.2.2.2 LncRNA and T-cells

It has been found that endometriosis patients have higher levels of TH17 than the control. Immediate early response gene (IER3) is one factor that drive the differentiation of T-cells to TH17. Bioinformatics analysis revealed that miR-342-3p, which is highly expressed in the serum of women with EMS, is a candidate microRNA for the 3’UTR of this gene. As lncRNA may control microRNAs through sponging, the lncRNA H19 was postulated and evaluated in the study of Zheying Liu et al. This research showed that along with reduced H19 levels, miR-342-3p expression level comes with a rise in concentration, then this microRNA binds to the 3′ UTR of IER3 and suppresses its expression. Suppression of IER3 ends up with a high level of TGF-β which increases RORγt; the transcription factor directs the differentiation of T-helper toward TH17 (121, 122).

##### 4.2.2.3 LncRNA and miscellaneous functions in the immunity

It is known that lncRNAs have additional functions in the immune system of EMS patients. Qing Yang found 500 lncRNAs with differential expression between the ectopic endometrial group and the control tissue, and 282 of these lncRNAs were associated with immunity. The results show that MIR202HG with proprotein convertase 2 (PCSK2/PC2) and AFAP1-AS1 with the tumor necrosis factor superfamily member 13 (TNFSF13) have a positive correlation. PCSK2 is an HLA-I-binding peptide linked with autoimmune diseases (123, 124). The TNF ligand superfamily member TNFSF13, which encodes the A proliferation-inducing ligand (APRIL), provokes memory B cells to undergo differentiation into plasma cells. In this way, AFAP1-AS1 may be a critical factor in the formation and maturation of B cells (124, 125). Furthermore, Tang et al. identified that the lncRNA AFAP1-AS1 was significantly correlated with PD-1 in nasopharyngeal carcinoma and that their coexpression predicted a poor prognosis (126). In endometriosis cancer studies, the expression of PD-1 on T cells has increased (127), considering that this is an exhaustion marker, using this lncRNA to reduce the PD-1 marker (128), as used in cancer immunotherapy, will be beneficial.

LINC02381 had never been reported in EMS before the research conducted by Meichen Yin. According to their investigation, this lncRNA can sponge hsa-miR-1301-3p, joining the 3’ UTR of CD84 (129). CD84 (SLAMF5) belongs to the signaling lymphocyte activation molecule (SLAM) family, and its mediated signaling regulates numerous immunological processes, which include natural killer cytotoxicity, T-cell cytokine secretion, monocyte activation, autophagy, T-cell B-cell interaction, and B cell tolerance at germinal center checkpoints (130, 131).

Gu et al. study determined the lncRNA and mRNA expression of patients with ovarian endometriosis via high-throughput RNA sequencing. After construction of the ceRNA network and functional enrichment analysis between the differentially expressed lncRNAs/mRNAs and miRNAs demonstrated that LINC01140, MSC-AS1, HAGLR, CKMT2-AS1, JAKMIP2-AS1, and AL365361.1 might act as an orchestrate in the positive regulation of myeloid leukocyte phagocytosis, and lymphocyte activation (132). **Table 2** shows up- and down-regulated lncRNAs in the endometriotic samples playing a role in the immune system response.

**Table 2:** differentional expressed LncRNAs having roles in immune system response in endometriosis.

LncRNA	Species	Sample	Assessed cell line	Target regulator	Mechanism	Study
**H19↓**	Human	PFMC from ovarian endometriotic cysts(n=20), control sample(n=16), PBMC from healthy fertile women, and endometrial tissues which are free in terms of EMS	_	miR-342-3p	Downregulation of H19 via negative regulation of the miR-342-3p/IER3 axis promotes TH17 differentiation and IL17 production. Additionally, H19 can regulate miR-17, an upstream regulator of TLR4.	(121)
**MALAT-1↑**	Human	Ectopic endometrial tissue (n=28), normal endometrial tissue(n=17)	_	miR-142-3p	Mediating inflammation by sponging miR-142-3p, an upstream regulator of CXCR7, and production of TNF-α, IL-1β, and IL-6.	(93)
**LINC00339↑**	Human	Endometrium and endometriotic lesion from EMS(n=251), control group(n=30)	T-HESC	DDX58, IFIH1, OAS2, OAS3, IFIT1, IFIT3, IFITM3, MX1, STAT1, STAT3, IFNE	Overexpression of LINC00339 causes Upregulation of PRRs and inflammation	(111)
**HOTAIR↑**	Human	Endometrial tissues sample from ovarian endometrial cysts(n=50), control sample(n=50)	_	miR-761	HOTAIR, via regulation of the miR-761/HDAC1 axis and activation of STAT3-mediated inflammation, takes part in the inflammatory response.	(114)
**LINC01018↑**	Human	GSE121406, GSE105764, GSE105765	_	hsa-miR-182-5p	Regulation of IFN-γ and TNF-α by sponging hsa-miR-182-5p	(115)
**LINC01272↑**	Human	GSE121406, GSE105764, GSE105765	_	hsa-miR-182-5p	Upregulation of LINC01272 regulates CHL1 by mediating hsa-miR-182-5p.	(115)
**CHL1-AS1↑**	Human	Ectopic endometrial tissue from ovarian EMS cysts(n=50), control sample(n=50), Peritoneal fluid	_	miR-610	CHL1-AS1 transported by exosomes enters eESCs, and by Sponging miR-610 led to the accumulation of MDM2 resulting proliferation, migration, and invasion of eESCs	(120)
**AFAP1-AS1↑**	Human	Paired ectopic and eutopic endometria(n=36)	_	TNFSF13	Have a role in B cells development and maturation.	(124)
**MIR202HG↑**	Human	Paired ectopic and eutopic endometria(n=36)	_	PCSK2/PC2	Modulating HLA-I binding peptide	(124)
**LINC02381↑**	Human	Normal endometria(n=6), eutopic endometria(n=6), and ectopic endometria(n=6)	_	hsa-miR-1301-3p	By sponging hsa-miR-1301-3p affects on CD84	(129)
**LINC01140↑, MSC-AS1↑, HAGLR↑, CKMT2-AS1↑, JAKMIP2-AS1↑, and AL365361.1↑**	Human	Ectopic endometria sample(n=8), eutopic endometria sample(n=8)	_	_	Positive regulation of phagocytosis in myeloid leukocyte and lymphocyte activation	(132)

### 4.3 CircRNA

Circular RNAs (circRNA) are a new type of noncoding RNA with a circular loop structure without a 5′ cap or a 3′ Poly A tail. Although it is not precisely known how circular RNAs in EMS work, new studies have revealed that they may play an essential role in eESC proliferation, migration, and invasion and could be used as molecular markers for the disease (133–135). In some studies, their regulatory roles in apoptosis and the cell cycle have also been examined (136). However, there have been minimal efforts to clarify circ RNA’s role in the immune responses in EMS.

#### 4.3.1 CircRNA and immune regulation

According to the research carried out by Xu et al., expression profiles of circRNAs were compared between ectopic and paired eutopic endometria and a circRNA-miRNA-mRNA network was established. The expression-related features of circRNA and mRNA in four patients were investigated using microarray, and eight circRNAs and mRNAs were verified using qPCR. The majority of the differentially expressed mRNAs were found to have a functional connection to immune-inflammatory responses and cell-cycle control. The qPCR findings for eight mRNAs and five circRNAs matched the microarray results (136). The evaluation of the ceRNA network indicated that ectonucleoside triphosphate diphosphohydrolase 1 (ENTPD1) is a target gene for the eESCs upregulated circ0004712 and circ0002198. CD39/ENTPD1 is the predominant ectonucleotidase which has a crucial immunomodulatory role in EMS tissue; downregulation of this gene in EMS tissue fails to maintain the tolerogenic immune function of Tregs and increases inflammation (137). Given that exosomes can transport ENTPD1 protein and the circRNAs (138, 139), the idea put forth is that the amount of this protein might be reduced in two ways. First, the exosomal transfer of circ0004712 and circ0002198 decreases the amount of ENTPD1 in Treg. Second, the suppression of this protein in ESCs by circRNAs resulted in a reduction in the exosomal transportation of ENTPD1 protein to immune-regulatory cells like Treg. Therefore, research in these areas needs studies of the effect of exosomal circ0004712 and circ0002198 derived eESCs in Tregs. Despite throughout evaluation of network and targets for mentioned circRNAs, the exact role of these ncRNAs has not been investigated in immune cells. Therefore, single cell studies which assess the physiological and pathological function specific cells, such as treg cells, after co-culturing them with eESCs and exposing them to external exosomes, can be useful.

#### 4.3.2 CircRNA and additional functions in the immune system

Another study that pointed to immune response dysregulation caused by circ RNA was by Meichen Yin et al. High-throughput sequencing revealed that 140 lncRNAs, 107 circRNAs, and 1,206 mRNAs were expressed differently in the ectopic group in comparison to the normal and eutopic groups. The Establishment of the ceRNA network was performed by differentially expressed miRNA, lncRNA, and circ RNA. Further analysis of the function of differential ncRNAs illustrated that these RNAs are related to the epithelial-mesenchymal transition, regulation of the immune system, and immune effector process.

circFN1, as an overexpressed circ RNA, was found to affect the expression of CD84, modulating a wide range of immune system processes by sponging has-miR-1301-3p in EMS (129). Generally, studies that employ high-throughput methods first identify differentially expressed ncRNAs and establish networks. Then, they analyze gene enrichment databases to gain insights into the pathways regulated by these RNAs. However, these databases provide only an overview of the pathways involved, requiring future studies to investigate the precise role of these ncRNAs in immune cells using *in vivo* models.

## 5 Conclusion

In recent years, ncRNAs have been at the center of attention in several medical research areas worldwide. According to research on both humans and animals, ncRNAs contribute to the pathophysiology of EMS. Although previous research typically only investigated the role of non-coding RNA in the regulation of cellular processes of ESCs. There has been minimal effort to use immune cells to determine their non-coding RNAs profile. For this reason, further research would be needed to determine the mechanism of these RNAs in the immune system. Taken together, mechanistically, ncRNAs can affect endometrial cells’ migration, invasion, proliferation, and apoptosis as well as the modulation of inflammation. They might potentially have an impact on lesion implantation. Furthermore, ncRNAs can alter fate and function of immune cells, including macrophages and NK cells. The alteration of immune cells can mutually promote inflammation. In addition to the role of ncRNAs in the immunopathogenesis of endometriosis, they can play a role in human health. Specifically, miR-21a and miR-16 are used as biomarkers in endometriosis diagnosis. Moreover, there is evidence that ncRNA inhibitors such as Let-7b can significantly prevent disease progression in animals. Thus, it is possible to utilize these inhibitors as part of a treatment approach for patients. A more inclusive evaluation of these transcripts by using the high throughput methods and discovery of the functional link between lncRNA/circRNA-miRNA-mRNA ceRNA network can give insights into the immunopathogenesis of EMS and identification of possible diagnostic and therapeutic targets in this regard.

## Author contributions

All authors listed have made a substantial, direct, and intellectual contribution to the work, and approved it for publication.
